# Inspired by an ancient Chinese Medicine prescription: the modern significance and potential of Yiyi Fuzi Baijiang San in treating diseases

**DOI:** 10.3389/fphar.2024.1465387

**Published:** 2024-11-07

**Authors:** Yuli Zhang, Lu Zhang, Ni Chai, ZhiQiang Wan, Hua Sui

**Affiliations:** ^1^ Department of Traditional Chinese Medicine, Jiading Branch of Shanghai General Hospital, Shanghai Jiao Tong University School of Medicine, Shanghai, China; ^2^ Medical Experiment Center, Jiading Branch of Shanghai General Hospital, Shanghai Jiao Tong University School of Medicine, Shanghai, China; ^3^ The Affiliated Cancer Hospital of Zhengzhou University and Henan Cancer Hospital, Zhengzhou, China; ^4^ Oncology Department, Yueyang Hospital of Integrated of Traditional Chinese and Western Medicine, Shanghai University of Traditional Chinese Medicine, Shanghai, China; ^5^ Jiading Branch of Shanghai General Hospital, Shanghai Jiao Tong University School of Medicine, Shanghai, China; ^6^ Center for Research and Graduate Studies, University of Cyberjaya, Selangor, Malaysia

**Keywords:** Yiyi Fuzi Baijiang San, traditional Chinese medicine, mechanism, active ingredients, clinical application

## Abstract

Classic Formulas (Jing fang) are considered the essence and authority of Traditional Chinese Medicine (TCM) due to their long history and proven efficacy. These formulas play a pivotal role in all kinds of different disease prevention and therapeutic strategies. Yiyi Fuzi Baijiang San (YYFZBJS), one of the Classic Formulas, was originally developed for the treatment of chronic intestinal abscess. With the accumulation of clinical experience and the exploration of modern pharmacological research in recent years, YYFZBJS has been extensively employed to address a broad spectrum of conditions such as colorectal cancer. Although numerous studies have explored the clinical efficacy and underlying mechanisms of YYFZBJS, no comprehensive review summarizing these findings exists to date. This study aims to systematically review and critically assess the current clinical and mechanistic research on YYFZBJS, with the objective of providing valuable insights and guidance for TCM research in the future.

## 1 Introduction

**FIGURE 1:**
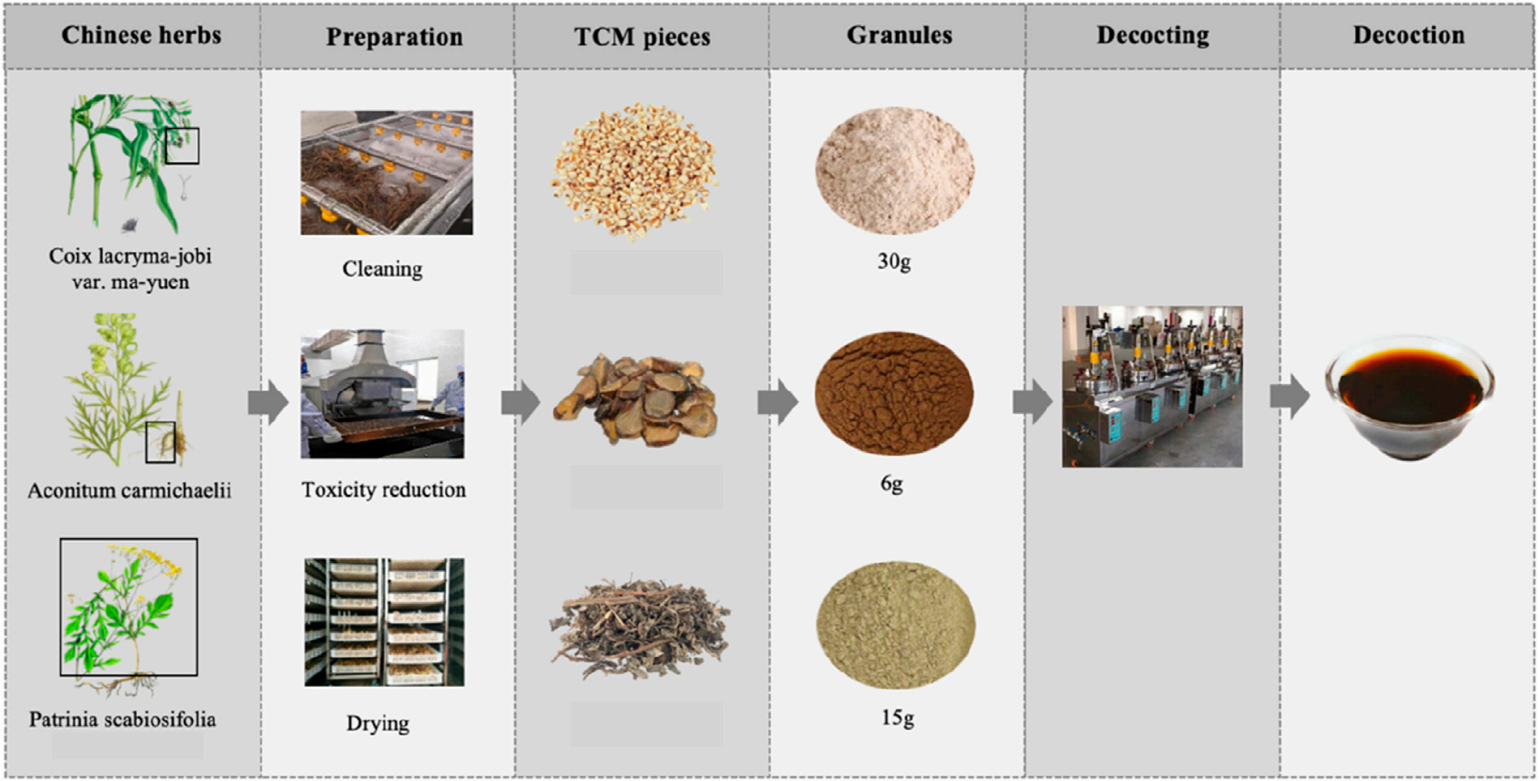
Preparation process of YYFZBJS.

Traditional Chinese Medicine (TCM), with its millennia-long history, is widely practiced in China and increasingly gaining recognition worldwide. Unlike modern medicine, TCM is distinguished by its approach to diagnosis and treatment, which emphasizes syndrome differentiation and holistic principles. By integrating clinical symptoms with an understanding of the body’s internal environment, TCM seeks to restore balance and address the underlying causes of illness. This holistic perspective informs its therapeutic strategies, providing a nuanced approach to prevention and treatment (Oravecz and Mészáros, 2012). YYFZBJS, one of the Classic Formulas first recorded in the ancient Chinese medical text *Synopsis of Prescriptions of the Golden Chamber*. It comprises three botanical drugs: *Coix lacryma-jobi* L. var. *mayuen* (Roman.) Stapf. (Coix seed), *Aconitum carmichaeli* Debx. (Radix Aconiti Lateralis), and *Patrinia villosa* (Thunb.) Juss. (Patrinia villosa). These botanical drugs undergo a process of washing, detoxification, drying, powdering, and are then decocted in the proportions of 30 g: 6 g: 15 g to produce a medicinal solution typically administered orally (Twice daily, 200 mL each time) (Figure 1). According to the original documentation, YYFZBJS was originally used to treat chronic intestinal abscess resulting from persistent pathogenic factors including yang deficiency, cold, dampness and blood stasis. With advancements in modern pharmacological research, the clinical applications of YYFZBJS are gradually expanding to various areas including tumor treatment. Previous research has elucidated several potential pharmacological mechanisms of YYFZBJS and its components in anti-inflammation, immune regulation, and disease prevention. Nevertheless, due to challenges such as its complex composition, the full scope of its clinical applications and underlying mechanisms remains unclear. This article provides a comprehensive review of its pharmacological effects and clinical applications, with the hope to deepen understanding and encourage further research on classical ancient prescriptions in TCM.

## 2 Review methodology

To investigate the clinical efficacy and underlying mechanisms of YYFZBJS, we conducted a comprehensive search of articles in PubMed, Web of Science, and China National Knowledge Infrastructure from January 2000 to May 2024. The following keywords were used: ‘Yiyi Fuzi Baijiang San,’ ‘Yiyi Fuzi Baijiang Powder,’ ‘YYFZBJS,’ ‘YYFZBJP,’‘Coix seed,’ ‘Adlay,’ ‘*C. lacryma-jobi* L. var. *mayuen* (Roman.) Stapf.’‘*Aconitum carmichaeli* Debx.’‘Radix Aconiti Lateralis,’‘Patrinia villosa,’‘*P. villosa* (Thunb.) Juss.’ ‘Traditional Chinese Medicine,’ ‘mechanism,’ and ‘clinical application.’ Articles published in peer-reviewed journals were considered. The retrieved articles were reviewed by two independent reviewers based on their titles, abstracts, and full texts, adhering to specific inclusion and exclusion criteria. The inclusion criteria were: 1) Original articles written in English or Chinese; 2) Articles that examined the relevant mechanisms or clinical application of YYFZBJS and the active components or herbs it contains. Exclusion criteria were as follows: 1) Articles written in any language other than English and Chinese; 2) Literature that is unrelated to YYFZBJS or its active components or herbs; 3) Editorials; 4) Conference abstracts; 5) Studies lacking adequate discussion on the review topic; 6) Duplicate publications.

## 3 Traditional applications of YYFZBJS

The botanical drugs comprising YYFZBJS each possess distinct and irreplaceable therapeutic properties, and their synergistic combination forms a solid foundation for the clinical application of YYFZBJS. Coix seed*,* the dried mature kernel of *C. lacryma-jobi* var. ma-yuen (Rom.Caill.) Stapf, is a widely utilized culinary and medicinal plant in Southeast Asia. In TCM theory, Coix seed is characterized by its cool nature, sweet and light flavour, and its association with the spleen, stomach and lung meridians (Hou J et al., 2018). It is widely recognized for its properties in clearing heat, expelling pus, relaxing tendons, relieving arthralgia, dispersing knots, strengthening the spleen, and dispelling dampness (Han X et al., 2017). Historically, Coix seed has been used to treat inflammatory conditions accompanied by abscesses. Radix Aconiti Lateralis*,* the lateral root tuber of *Aconitum carmichaeli* Debx*,* is worm in nature, pungent and sweet, and associated with the heart, kidney and spleen meridians. It is important to note that Radix Aconiti Lateralis must undergo standardized and meticulous processing before it can be safely applied in clinical practice (Yang M et al., 2018). Secondly, Radix Aconiti Lateralis is known for its functions in restoring yang, relieving collapse, tonifying fire, dispelling cold, and alleviating pain. Over time, its clinical applications have expanded to include the treatment of various conditions such as cancer, heart failure, colitis, and rheumatoid arthritis (Fu Y et al., 2022; Tai C et al., 2021). Thirdly, Patrinia villosa is cool in nature, pungent and bitter in taste, and is linked to the liver, stomach and large intestine meridians. As a classical heat-clearing and detoxifying herb, Patrinia villosa is used to eliminate carbuncles, expel pus, dispel blood stasis, and relieve pain. Consequently, it is commonly employed in treating intestinal abscess, lung carbuncles, gynecological epigastric pain, *postpartum* blood stasis, and eczema (He et al., 2019; Gong et al., 2021).

## 4 Active ingredients of YYFZBJS

**FIGURE 2:**
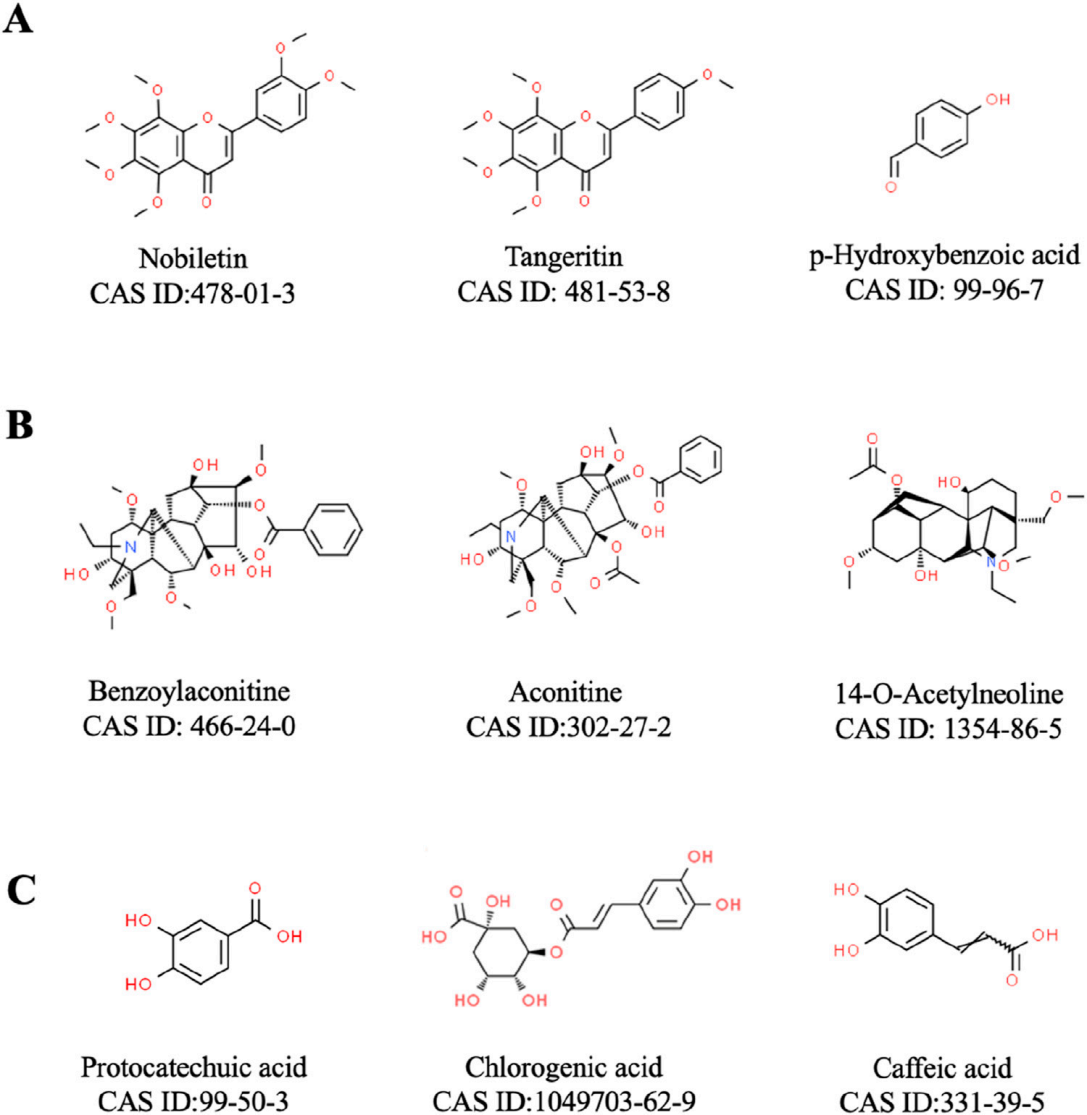
Representative metabolites included in YYFZBJS. **(A)**: Coix seed; **(B)** Radix Aconiti Lateralis; **(C)** Patrinia villosa).

Analyzing the active ingredients of YYFZBJS is essential for understanding its pharmacological mechanisms, as it facilitates the identification of key bioactive compounds and their interactions with various targets. Advanced analytical techniques, such as UPLC-MS, have been employed to identify several significant ingredients in YYFZBJS, including liquiritigenin, aconitine, hypaconitine, luteolin, and puerarin (Zhang et al., 2022). Furthermore, additional components such as Coix seed oil (CSO), flavonoids, and Aconitum alkaloids have also been isolated from YYFZBJS (Table 1; Figure 2). Beyond merely detecting these ingredients, network analysis methods play a pivotal role in clarifying the synergistic effects and mechanisms underlying the multi-component, multi-target nature of YYFZBJS. These methods help in mapping the complex interactions among the bioactive compounds and their respective targets, providing a comprehensive understanding of how the formula exerts its therapeutic effects (Sui et al., 2020). This approach has revealed the diverse biological activities of YYFZBJS, highlighting its anti-inflammatory, anti-cancer, and antioxidant properties (Sun et al., 2020; Fang et al., 2018a). Such integrated analyses provide researchers with valuable insights into the therapeutic potential of YYFZBJS and its potential clinical applications.

**TABLE 1:** Botanical drugs and components contained in YYFZBJS.

TCM drugs	Family	Original plants	Medicinal parts	TCM application	Authenticated metabolites		References
Coix seed	Poaceae	*Coix lacryma-jobi* L. var. *mayuen* (Roman.) Stapf	dried mature seed kernel	promoting urination and draining dampness, invigorating spleen, clearing heat and expelling pus, relieving impediment	polysaccharides	PAS-1, PAS-2, PAS-3, PAS-4, Fructooligosaccharides	Chinese Pharmacopoeia commission (2020), p.393; Li *et al.* (2020)
					Fatty acids and esters	trilaurin, 3-octadecoxypropane-1,2-diol, 2-dimethylaminoethyl tetradecanoate, Palmitic acid-13C, palmitic acid, azelaic acid, Stearic acid-1–13C, oleic acid, linoleic acid	
					Amino acids	valine, leucine, glutamic acid, argininic acid, phenylalanine	
					Polyphenols	hydroxybenzoic acid, vanillic acid, eugenol, ferulic acid, p-coumaric acid, caffeic acid, mustardic acid, vanillic acid, 2-hydroxyphenylacetic acid, barley alcohol, 4-ketopineol ester, eugenol, catechuic acid	
					Sterols	α-sitosterol, β-sitosterol, γ-sitosterol, rape sterol, ergosterol, cholestrol, obtuse leaf macrostanol, feruloylsitosterol, brassinosteroid, soya sterol	
					Flavonoids	quercetin, kaempferol, rutin	
					Endocannabinoids	coixol	
					Triterpenoids	friedelin, cylindrin	
Radix Aconiti Lateralis	Ranunculaceae	*Aconitum carmichaeli* Debx	Lateral root tuber	restoring yang to save from collapse, tonifying fire and assisting yang, dissipating cold and relieving pain	Alkaloids	benzoylaconine, benzoylmesaconine, beiwutine, 14-O-cinnamoylneoline, 14-O-acetylneoline, 14-O-anisoylneoline, 14-O-veratroylneoline, bulleyaconitine A, lipomesaconitine, lipo-14-O-anisoylbikhaconine, lipo-14-O-anisoylbikhaconine, mesaconine	Chinese Pharmacopoeia commission (2020), p.200; Rong *et al.* (2021)
					Saponins	gracillin	
					Ceramides	(2S,3S,4R,8E)-2-[(2′R)-2′-hydroxylignoceroylamino]-8 (E)-octadecene-1,3,4-triol	
					Volatile oils	palmitic acid, 1-palmitoleoyl glycerol, tridecylic acid, linoleic acid	
					Others	β-sitosterol, daucosterol, uracil, adenosineetc.	
Patrinia villosa	Caprifoliaceae	*Patrinia villosa* (Thunb.) Juss	Whole plant	clearing heat and removing toxin, eliminating mastitis and expelling pus, dispelling stasis and relieving pain	Phenylpropanoids	caffeic acid, ferulic acid, caffeic acid ethyl ester, trans-ferulic acid, trans-caffeic acid methylate, chlorogenic acid n-butyl ester, chlorogenic acid butyl ester, scopoletin, gallic acid, 5-methoxyisolariciresinol, 7R,8S-glochidioboside, tanegool, pinoresinol, massonianoside D, interosode B, lyoniresinoletc.	He *et al.* (2017), Gao *et al.* (2011b), Peng *et al.* (2006), Fan *et al.* (2022)
					Flavonoids	Luteolin-6-C-glucoside, Isovitexin, tetrapterol I, luteolin, 3′-prenyl-apigenine, apigenin、kaempferol-3-O-β-D-galactopyranoside, kaempferol-3-O-rhamninoside, bolusanthol B, patriniaflavanone A, catharticin, kaempferol-3-O-trirhamninoside, rutin, quercetin, puerarin, orotinin, orotinin-5-methyl ether, 5,7,2′,6′-tetrahydroxy-6,8-di (γ,γ-dimethylallyl) flavanone, 5,7,2′,4′-tetrahydroxy-8,3′-di (γ,γ-dimethylallyl)-isoflavanoneetc.	
					Phenols	1-O-(β-D-glucosyl)-2-[2-methoxy-4-(3-hydroxypropyl)-phenoxy]-propan-3-ol, methyl 2-(4-hydroxyphenyl) acetate, 4-ethyl-phenol, resorcinol, dihydrosinapyl alcohol, 2-methoxy-1,3-benzenediol	
					Terpenoids	limonene, linolenic acid, methyl ester, perillaldehyde, patrinialactones A, patrinialactones B, loliolide, isololiolide, sphenanthin A, perilla alcohol, sweroside, villosol, villosolside, palmiticacid, loganin, morroniside, villoside, patrinalloside, valerosidate, loganic, sweroside, villosol, villosolside, palmitic acid, loganin, morroniside, villoside, patrinalloside, valerosidate, loganic acid, patrinovalerosidate, patrinoside-aglucone acid, patrinovalerosidate, patrinoside-agluconeetc.	
					Steroids	7β-hydroxysitosterol, stigmasterol, pubinernoid A, daucosterol, β-daucosterol, spinasterol	
					Alkaloids	urea, aurentiamide acetate, 1H-indole-3-carbaldehyde, N-benzoylphenylalanyl-L-phenylalaninol acetate, 3S,4R-(+)-4-hydroxymelleinetc.	
					Organic acids	protocatechuic acid, chlorogenic acid, linoleic acid, undecanoic acid, tetradecanoic acid, pertedecanoic acid, 3-methyl-butanoic acid, tanninetc.	
					Aromatic metabolites	Inositol, phenol, o-cresol, p-cresol, dimethoxyphenol, 2-ethylphenol, 4-ethylphenol, p-hydroxybenzoic acid, ent-eudesm-4 (15)-ene-1β,6α-dioletc.	
					Volatile oils	menthalignin, (−)-camphor, borneol, ethyl myristate,6-aminoisoquinoline, n-hexanal, heptanal, isocentdarol, 6-aminoisoquinoline, linalyl butyrate, cis-thujone, menthol, 2,6-dimethoxyphenol, 1-chloroheptane, 2-methyl-6-ethyldecane, methyl myristateetc.	

## 5 Pharmacological activities of YYFZBJS and its constituents

The clinical application of YYFZBJS is strongly supported by an abundance of in-depth modern pharmacological research. Although the content of constituents in YYFZBJS decoction liquid may vary with different extraction methods, the active components with high content are mainly CSO and flavonoids, and their pharmacological effects have been continuously recognized (Zhou et al., 2016).

### 5.1 Signaling pathway regulation in inflammation and metabolism

**FIGURE 3:**
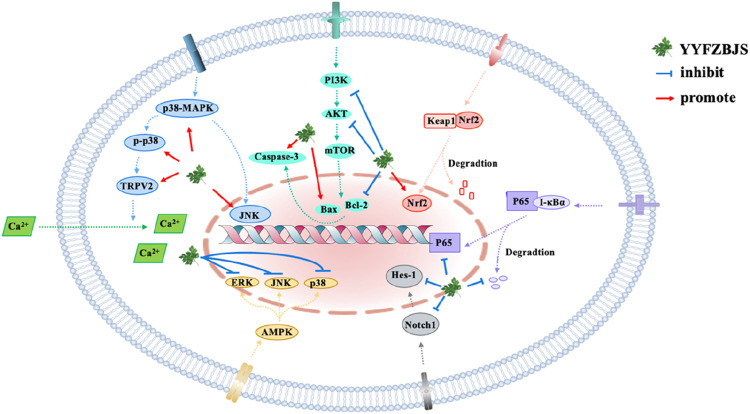
Signaling pathways affected by YYFZBJS.

The crucial role of signaling pathways in metabolic disorders and infections lies in their influence on cellular responses. These pathways are intricately linked to various cellular processes, including metabolism, gene expression, alterations in intracellular enzyme activities, cytoskeletal structure, and DNA synthesis. In keeping with these, numerous studies have demonstrated that YYFZBJS plays a therapeutic role in modulating specific signaling pathways (Figure 3).

For instance, the Nrf2 signaling pathway is recognized as one of the most essential mechanisms in the regulation of antioxidant stress responses. YYFZBJS has been shown to increase the expression of Nrf2 and its downstream antioxidant protein HO-1 in rodents with UC, resulting in a decreased inflammatory response (Fang et al., 2018b). However, the precise metabolites responsible for this effect remain unidentified.

One of the principal active components of YYFZBJS, CSO, has been reported to modulate signaling pathways involved in lipid metabolism (Qin et al., 2023). Studies have identified that CSO primarily consists of oleic acid (50.54%), linoleic acid (33.76%), palmitic acid (11.74%), and stearic acid (2.45%), with its fatty acid profile closely matching that of vegetable oil (Ni et al., 2021). CSO has been shown to influence the p-AMPK/SePP1/apoER2 signaling pathway, which regulates lipid accumulation in liver tissue. Researchers demonstrated that CSO could inhibit the phosphorylation of adenosine 5′-monophosphate (AMP)-activated protein kinase (AMPK), leading to a reduction in the expression of SePP1/apoER2, thereby decreasing lipid accumulation, as observed in both in vivo and in vitro experiments (Gu et al., 2021). Similarily, Benzoylaconitine, another active metabolite of YYFZBJS, has emerged as a potential therapeutic agent for synovial inflammation in rheumatoid arthritis. The underlying mechanism appears to involve the inhibition of the PI3K/Akt signaling pathway, through the suppression of IL-1-induced expression of IL-6 and IL-8, inflammatory cytokines associated with rheumatoid arthritis (Yu et al., 2020). Additionally, Benzoylaconitine targets the MAPK and NF-κB signaling pathways to exert its anti-inflammatory effects. In summary, the regulation of signaling pathways is a critical mechanism by which YYFZBJS exerts its therapeutic effects.

### 5.2 Gene and protein modulation in tumor and muscle atrophy

Researchers have identified that the active metabolites of YYFZBJS have the potential to regulate gene and protein expression, which is crucial in addressing the abnormal expression contributing to the onset and progression of various diseases. For instance, Chen *et al.* discovered that CSO significantly upregulated the expression of stress-inducible genes, such as *daf-16*, *sod-3*, *hsp-16.2*, and *gst-4* in *Caenorhabditis elegans* (Chen X. Y. et al., 2020). In another study, CSO was shown to increase the binding of the NF-κB p65 subunit to the promoter regions of IL-2- and Bcl-2-encoding genes in tumor-bearing mice (Huang X. et al., 2014). Radix Aconiti Lateralis has also been found to exert therapeutic effects through the modulation of protein expression. The specific mechanisms include the significant suppression of dexamethasone-induced mRNA expressions of muscle atrophy F-box protein (MAFbx)/atrogin1, Casitas B-lineage lymphoma-b (Cbl-b), troponin, and branched-chain amino acid aminotransferase 2 (BCAT2), thereby inhibiting muscle atrophy (Kondo et al., 2022). Additionally, Aconitum alkaloids, the main metabolites of Radix Aconiti Lateralis, have been observed to increase the expression of multidrug resistance-associated protein 2 (MRP2), providing insights into the clinical application of plants in the Aconitum family (Wu et al., 2018). It is well recognized that tumor patients are prone to muscle atrophy and weakness as their illness progresses; however, the role of YYFZBJS’s active metabolites in treating muscular dystrophy in these patients remains unclear and warrants further exploration and validation (Williams et al., 2021).

### 5.3 Apoptosis-based therapeutic and potential toxic effects

Apoptosis is a genetically regulated process by which cells autonomously undergo programmed death, playing a crucial role in maintaining a stable internal environment. Mitochondrial proteins such as Bcl-2, Bax, and Cyt-c are activated and oligomerize on the outer mitochondrial membrane, mediating its permeability-a critical step in the apoptotic process. One study found that CSO can regulate mitochondrial apoptotic pathways by downregulating Bcl-2 and upregulating Bax, cleaved caspase-9, cleaved caspase-3, and Cyt-c proteins (Yang et al., 2022). Another active metabolite, SPVJ, extracted from Patrinia villosa, has been reported to significantly increase the number of apoptotic cells (from 9.42% to 28.9%) in U14 cervical cancer-bearing mice when administered at a dose of 100 mg/kg body weight (p.o.), compared to the control group receiving distilled water (p.o.) (Zhang et al., 2008). Additionally, Aconitum alkaloids have been demonstrated to induce apoptosis in various tumor cells without affecting normal cells, as confirmed by several studies (Fan et al., 2016; Qu et al., 2020). However, it is important to note that apoptosis is also implicated in the cardiotoxicity and neurotoxicity associated with aconitum plants. Xia *et al.* conducted a developmental toxicity assay of Aconitine on zebrafish embryos and found that high doses (7.27 and 8.23 μM) of Aconitine increased the levels of reactive oxygen species (ROS) and induced apoptosis in embryonic heart and brain regions (Xia et al., 2021). Furthermore, Aconitine was found to promote intracellular Ca2^+^ accumulation and cardiomyocyte apoptosis through the p38 MAPK signaling pathway in a dose-dependent manner (Yang et al., 2021). These findings suggest that apoptosis is not only related to the therapeutic effects of Aconitine but also to its potential toxicity. Therefore, the anti-apopotic function of YYFZBJS may be achieved through the regulation of mitochondrial proteins, remodeling ion channels, and activating signaling pathways.

### 5.4 Modulation of immune response for diverse effects

**FIGURE 4:**
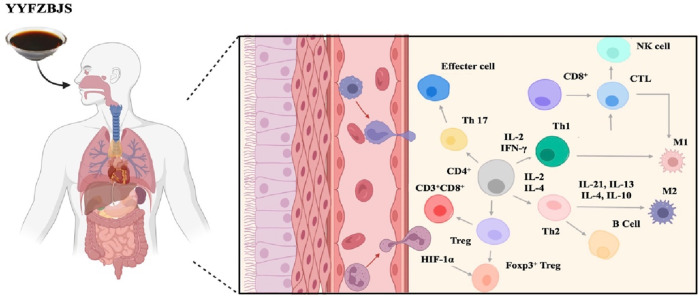
Immune cells affected by YYFZBJS.

As is well known, the immune system plays a crucial role in defending the body against pathogens and maintaining overall health by identifying and neutralizing harmful microorganisms and abnormal cells. Along with the development in molecular biology and immunology, the mechanism of the therapeutic effect on YYFZBJS has improved quickly in recent years. (Figure 4).

It is widely recognized that regulatory T (Treg) cells can inhibit anti-tumor immune responses, thereby facilitating tumor progression and invasion. Preliminary studies conducted by our research team have confirmed that the effects of oral administration of YYFZBJS on azoxymethane (AOM)/dextran sulfate sodium (DSS)-induced tumorigenesis in C57BL/6J mice. YYFZBJS treatment was found to reduce tumor load, tumor number, histological severity, and disease activity index (DAI) scores. Additionally, the study observed that the tumor-inhibiting effects of YYFZBJS were diminished in a Treg-deficient mouse model, compared to mice treated with YYFZBJS alone. This suggests that the enhanced immune response mediated by peripheral Tregs (pTregs) plays a crucial role in the anticancer activities of YYFZBJS (Sui et al., 2020; Zhang et al., 2022). The immune-regulating effects of YYFZBJS are closely related to its herbal components, as confirmed by another clinical study where Coix seed was the main intervention method (Jinnouchi et al., 2021). In animal models, the ethyl-acetate fraction of the ethanolic extract of Coix seed (ABE-EtOAc) has been shown to treat reversible increases in Th1/Th2 immunity by upregulating the expression of IL-2 and IL-4, reducing the release of histamines and cytokines such as IL-6 and TNF, and decreasing Akt production (Chen et al., 2012a; Chen et al., 2012b). Additionally, alkali-extractable polysaccharides from Coix seed have been shown to stimulate the production of molecules such as NO, TNF, and IL-6 in RAW264.7 murine macrophages a dose-dependent manner (Yao et al., 2015). Moreover, strong evidence indicated that the water-soluble polysaccharide fractions of Radix Aconiti Lateralis can stimulate lymphocyte proliferation, enhance antibody production, and promote macrophage phagocytosis, thereby boosting immune responses in the host (Zhao et al., 2006; Gao T. et al., 2011).

### 5.5 Therapeutic effects via gut microbiota modulation

**FIGURE 5:**
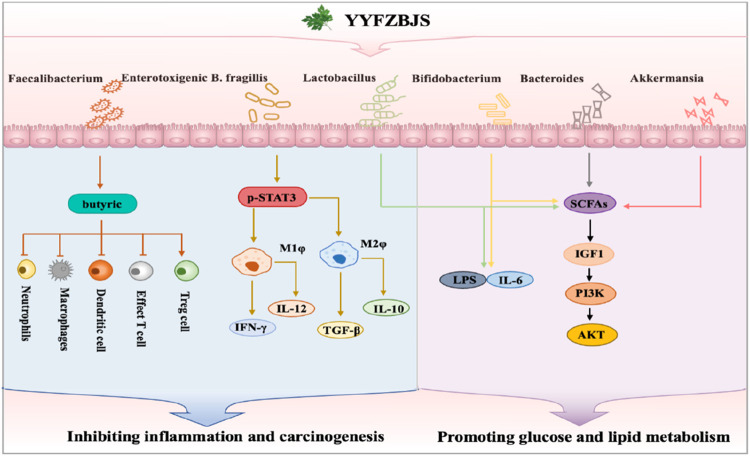
Intestinal flora affected by YYFZBJS.

An increasing number of studies are highlighting the role of TCM formulas in modulating the microbiota, reflecting growing interest in their impact on microbial balance and health (Hwang et al., 2016). It is well-established that the intestinal flora represents a complex microecosystem involved in numerous pathological processes, which can lead to diseases such as inflammatory bowel disease (IBD), cancer, diabetes, and tumors (De Vos et al., 2022). Modern pharmacological research has demonstrated that YYFZBJS possesses remarkable properties in modulating the intestinal flora, thereby exerting therapeutic effects on various diseases (Figure 5). Our previous studies revealed that the intestinal flora of *APC* ^*Min/+*^ mice treated with YYFZBJS exhibited significant alterations, including an increase in beneficial bacterial species such as Bifidobacterium and a decrease in harmful species such as *Bacteroides*, norank_f_ Erysipelotrichaceae (Zhang L. et al., 2020). The dysbiosis caused by enterotoxigenic *Bacteroides fragilis* has also been found to contribute to the development of colorectal cancer (CRC) by activating the p-STAT3 receptor, which influences M2 macrophage polarization. However, YYFZBJS can inhibit this process, thereby preventing chronic inflammation and the malignant transformation of adenomas (Chai et al., 2021). Coix seed was found to promote the spontaneous regression of viral cutaneous infections in healthy adult males by increasing the abundance of gastrointestinal Faecalibacterium (Li et al., 2021b). Additionally, the hypoglycemic efficacy of polysaccharides from Coix seed was also demonstrated through their ability to modulate the gastrointestinal microbiota, specifically by increasing the production of short-chain fatty acid (SCFA)-producing bacteria (Ge et al., 2024).

## 6 Clinical application and therapeutic potential of YYFZBJS

### 6.1 Clinical efficacy in the management of inflammatory diseases

Traditionally, YYFZBJS was used to treat chronic intestinal abscesses, which are localized inflammations of the intestines. However, with the accumulation of clinical experience and the advancement of pharmacological research, YYFZBJS and its constituent herbs have been proven to possess broader anti-inflammatory effects, demonstrating significant efficacy in treating various intestinal and extra-intestinal diseases.

#### 6.1.1 Decoction of YYFZBJS

The anti-inflammatory properties of YYFZBJS are primarily attributed to its constituents, Patrinia villosa and Coix seed. With the adjunctive support of Radix Aconiti Lateralis, YYFZBJS is more widely utilized in the management of chronic inflammatory diseases, including UC, Crohn’s disease, and anal sinusitis. Notably, YYFZBJS is typically administered via oral ingestion or retention enemas as a novel approach to the UC treatment, and the clinical efficacy has been confirmed in randomized controlled trials involving modest sample sizes (Hu et al., 2020; Li and Chen, 2021; Lv and Zhang, 2017; Zhang S. X. et al., 2019; Liu et al., 2023). Some clinical trials also demonstrated that patients receiving YYFZBJS have a higher effective rate and a lower recurrence rate compared to those treated with antibiotics such as norfloxacin and metronidazole for anal sinusitis (Wei et al., 2013; Guo et al., 2009). In addition, YYFZBJS is often combined with other treatments for inflammatory diseases. Single-center randomized controlled studies have shown that combining YYFZBJS with other TCM prescriptions, such as Dachaihu Decoction or Guizhi Fuling Pills, can significantly alleviate the clinical symptoms of chronic prostatitis with few adverse effects and reduced costs (Kong, 2016;Liu and Liu, 2018). Furthermore, it is reported that YYFZBJS may be effective in treating chronic gynecological inflammatory diseases like chronic pelvic inflammatory disease (PID) and chronic skin conditions like acne (Wu et al., 2016; Zhang and Chen, 2021). Overall, YYFZBJS has broad applications in the treatment of inflammatory diseases, with one of its most prominent advantages being its ability to significantly reduce the recurrence of chronic infectious diseases.

#### 6.1.2 Constituents in YYFZBJS

Recent studies have elucidated that the anti-inflammatory mechanisms of YYFZBJS are closely associated with its constituent metabolites. Coix seed, the botanical component with the highest concentration in YYFZBJS, exhibits significant anti-inflammatory properties. Notably, ethyl acetate extracts of Coix seed have been found to contain anti-inflammatory flavonoids and phenolic compounds, such as tangeretin, nobiletin, and p-hydroxybenzoic acid. These compounds are thought to exert their effects through potent inhibition of NO production, suppression of inducible nitric oxide synthase (iNOS) and cyclooxygenase-2 (COX-2) expression, and reduction of pro-inflammatory cytokines, IL-6 and TNF-α (Huang D. W. et al., 2014; Seo et al., 2000; Huang et al., 2009).

Additionally, other metabolites within YYFZBJS demonstrate substantial anti-inflammatory effects. 14-O-acetylneoline, a diterpenoid alkaloid derived from Radix Aconiti Lateralis, has been shown to protect rodents from various forms of intestinal inflammation by reducing IFN-γ production (Wangchuk et al., 2015). In an *in vivo* study, colonic IFN-γ mRNA levels were significantly lower in mice treated with 14-O-acetylneoline compared to control mice administered trinitrobenzoylsulfonic acid. Although further research is necessary, this alkaloid is currently considered a promising candidate for the development of anti-colitis therapeutics. Moreover, hydroalcoholic extracts of Radix Aconiti Lateralis have been reported to inhibit dense inflammatory cell infiltration in the lamina propria of animals with gastric ulcers by mitigating detrimental free radical cascades and oxidative stress (Rajakrishnan et al., 2020). The anti-inflammatory effect of Patrinia villosa is equally noteworthy. Methanol extracts of Patrinia villosa roots have demonstrated anti-inflammatory effects at colorectal sites by inhibiting NF-κB p65 activation and reducing levels of inflammatory mediators such as IL-6 and TNF (Cho et al., 2011; Lee et al., 2012). In rodent models of PID, Patrinia villosa significantly reduced inflammatory cell infiltration in the pelvis. The underlying mechanisms may involve the downregulation of lactate dehydrogenase and pyruvate carboxylase, coupled with the upregulation of arachidonic acid esters (Zou et al., 2015). Furthermore, Patrinia villosa has been reported to mitigate cutaneous inflammation in rodents with atopic dermatitis by increasing filaggrin expression and reducing inflammation-related cytokines and IgE levels, potentially through inhibition of JNK1/2 phosphorylation (Cha et al., 2017).

### 6.2 Clinical efficacy in the management of cancer

Recent research has increasingly highlighted the link between inflammation and various stages of cancer, including its onset, progression, and recurrence (Wang et al., 2024). This recognition underscores the importance of inflammation inhibition as a critical strategy in cancer treatment. With an understanding of the close relationship between inflammation and tumorigenesis, the potential anti-cancer effect of YYFZBJS has also become a research topic of significant interest (Table 2).

**TABLE 2:** Antitumor effects of YYFZBJS and its ingredients.

TCM	Ingredients	Cancer	Types of experiments	Effect	References
YYFZBJS	Decoction	CRC	Clinical trial	Advance the time for oral intake in patients after CRC surgery and facilitate the healing of surgical incisions	Zhang *et al.* (2020a)
YYFZBJS	Decoction	CRC	Clinical trial	Reduce the levels of CEA and CA19-9 in patients with CRC	(Nie 2020)
YYFZBJS	Decoction	CRC	*In vivo* (C57BL/6 J mice)	Regulate the polarization of peripheral Treg to suppress CRC cell proliferation and infiltration	(Zhang et al., 2022)
Coix seed in YYFZBJS	Ethanolic extract	CRC	*In vivo* (F344 rats)	Reduce the number of preneoplastic aberrant crypt foci and modified their mucin composition	(Li et al., 2011)
Coix seed in YYFZBJS	Coix seed oil	Pancreatic cancer	Clinical trial	Improve progression-free survival and quality of life	(Schwartzberg et al., 2017)
Coix seed in YYFZBJS	Coix seed oil	Lewis lung carcinoma	*In vivo* (Kunming mice)	Inhibit tumor growth and increase the spleen index	(Duan 2018)
Coix seed in YYFZBJS	Coix seed oil	Gastric cancer	Clinical trial	Alleviate gastrointestinal reactions and bone marrow suppression	(Zhan et al., 2012)
Patrinia villosa in YYFZBJS	Ethanolic extract	CRC	*In vitro* (HCT-8 cells)	Inhibit drug resistance in CRC cells	(Zhou et al., 2018)
Patrinia villosa in YYFZBJS	Giganteaside D	Hepatocellular carcinoma	*In vitro* (HepG2 cells, Bel-7402 cells)	Induce ROS-mediated apoptosis in HCC-derived cells	(Liu et al., 2016)
Patrinia villosa in YYFZBJS	Patrinia monoterpene iridoid ether esters	Hepatocellular carcinoma, Breast cancer	*In vitro* (HepG2 cells, MCF7 cells)	Inhibit tumor cell proliferation and induce apoptosis	(Ji et al., 2019)
Radix Aconiti Lateralis in YYFZBJS	Amide alkaloid	Hepatocellular carcinoma	*In vitro* (SMMC-7721 cells)	Induce apoptosis and cell cycle arrest in S phase	(Zhang et al., 2018)

#### 6.2.1 Multiple cancers including colorectal cancer

TCM has traditionally emphasized the observation and summary of disease symptoms, which complicates the identification of a specific diagnosis directly corresponding to CRC. However, the characteristic clinical manifestations of CRC suggest a close association with the TCM concept of “intestinal abscess,” which is characterized by severe symptoms and treatment difficulties, and relevant to the indications for YYFZBJS (Liu, 2017).

Clinical studies have indicated that the combination of YYFZBJS with chemotherapy may improve treatment outcomes for CRC and facilitate postoperative recovery. Patients with postoperative CRC who received YYFZBJS experienced significantly shorter times to first defecation, first bowel movement, and initiation of oral feeding (*p* < 0.05), along with notably higher plasma albumin levels compared to those who did not receive YYFZBJS (Nie, 2020; Zhang Y. et al., 2020). Additionally, administration of Coix seed and its ethanolic extract in rodent models has been shown to reduce preneoplastic aberrant crypt foci (ACF) and alter mucin composition, potentially preventing colonic preneoplastic lesions (Li et al., 2011). Preliminary research also suggests that Patrinia villosa may decrease the resistance of intestinal cancer cells to 5-fluorouracil (5-FU), indicating that it could enhance the effectiveness of CRC chemotherapy (Zhou et al., 2018).

Beyond CRC, emerging evidence suggests that YYFZBJS and its constituent botanical drugs may also be beneficial in other cancer types *(* Han et al., 2019; Song et al., 2018; Hu et al., 2023). A randomized, open-label study on pancreatic cancer patients demonstrated that Kanglaite (a pharmaceutical-grade emulsion of Coix seed oil) combined with gemcitabine significantly improved progression-free survival compared to gemcitabine alone (Schwartzberg et al., 2017). The addition of Coix seed to chemotherapy regimens for lung and gastric cancers has also yielded positive outcomes (Duan, 2018; Zhan et al., 2012). The therapeutic efficacy of Coix seed may be attributed to its high solubility and bioavailability. Coix seed oil (CSO), due to its unique texture and anti-tumor effects, has been developed into an oil phase component of microemulsions for anti-tumor treatment, showing promising results in breast, cervical, and lung cancers (Qu et al., 2017b; Chen Y. et al., 2020; Qu et al., 2017a). Moreover, Kanglaite alone has been reported to alleviate cancer-related pain and improve the quality of life for cancer patients (Zhang P. et al., 2019).

#### 6.2.2 Antitumor potential of the constituents in YYFZBJS

As a crucial component of YYFZBJS, Radix Aconiti Lateralis has demonstrated significant potential in anti-tumor activity. The diterpene alkaloids, which are categorized into C18-, C19-, C20-, and bis-diterpenoid alkaloids, are the principal components responsible for its therapeutic effects. These compounds exhibit notable cytotoxicity against various tumors, including lung cancer, prostate cancer, and triple-negative breast cancer (Wada and Yamashita, 2019; Thawabteh et al., 2021). In addition to the naturally alkaloids extracted from Radix Aconiti Lateralis, synthetic alkaloids derived from its metabolites have also demonstrated significant antitumor activity. For instance, ITPD, a metabolite from Radix Aconiti Lateralis, exerts its antitumor effects by activating caspase-3 and caspase-9, inducing Bax/Bcl-2 imbalance, leading to DNA damage and subsequent cell apoptosis (Zhang et al., 2018). In Patrinia villosa, the main antitumor components identified include polysaccharides, giganteaside D (GD), and Patrinia monoterpene iridoid ether esters (PMIEE), which has been reported to inhibit cancer cell proliferation and induce apoptosis in liver, breast, and cervical cancer cells through the downregulation of Bcl-2, CDC2, and Cyclin B1, and the upregulation of Bax and caspase-3 (Gong et al., 2021; Ji et al., 2019; He et al., 2019). Additionally, GD has been found to induce reactive oxygen species (ROS) production, leading to mitochondria-mediated apoptosis in hepatoma cells, and its cytotoxicity is associated with modulation of the MAPK signaling pathway (Liu et al., 2016; Xie et al., 2017).

## 7 Conclusion and perspectives

The use of YYFZBJS has a history spanning over 1800 years, but the concept of evidence-based medicine is relatively recent. As a result, there is currently limited support from large-scale clinical studies and historical data. Furthermore, the broad clinical application of YYFZBJS, as discussed in this study, is not arbitrary or widespread. Aligned with the TCM principle of pattern identification and treatment, YYFZBJS is primarily used to treat patients with a cold-heat complex pattern as determined by competent TCM practitioners.

As highlighted in this review, the exact ingredients of YYFZBJS have not yet been identified, and the exact action mechanisms of YYFZBJS are still unclear. Therefore, extensive research and rational standardization are crucial for the successful clinical application and promotion of YYFZBJS. Additionally, more clinical trials and cohort studies are needed to establish the therapeutic benefits of these herbs.

## Glossary

TCM: traditional Chinese medicine
YYFZBJS: Yiyi Fuzi Baijiang San
CSO: Coix Seed Oil
Nrf2: Nuclear Factor erythroid 2-Related Factor 2
HO-1: heme oxygenase 1
Bax: BCL2-associated X protein
Bcl-2: B-cell lymphoma/leukemia-2 gene
PI3K: Phosphoinositide-3 kinase
MAPK: mitogen-activated protein kinase
NF-κB: nuclear factor kappa-B
AMPK: Adenosine 5‘-monophosphate -activated protein kinase
SePP1: selenoprotein
P: plasma, 1
apoER2: apolipoprotein E receptor 2
MAFbx: muscle atrophy F-box protein
Cbl-b: Casitas B-lineage lymphoma-b
BCAT2: branched-chain amino acid aminotransferase 2
MRP2: multidrug resistance-associated protein 2
ROS: reactive oxygen species
UC: Ulcerative colitis
INOS: Inducible Nitric Oxide Synthase
COX: Cyclooxygenase
JNK: c-Jun N-terminal kinase
CRC: colorectal cancer
HIF-1α: hypoxia inducible factor-1α
ACF: preneoplastic aberrant crypt foci
EMT: epithelial-mesenchymal transition
TNF: tumor necrosis factor
PMIEE: Patrinia monoterpene iridoid ether esters
PID: Pelvic inflammatory disease
NAFLD: Nonalcoholic Fatty Liver Disease
TC: total cholesterol
**TG**: triacylglycerol
HDL: high-density lipoprotein
RyR2: Rabbit Ryanodine receptor 2
SERCA: sarco/endoplasmic reticulum Ca^2+^-ATPase
LPC: lysophosphatidylcholine
Treg: regulatory T cell
PCNA: proliferating cell nuclear antigen
GSH-Px: Glutathione peroxidase
CAT: Catalase
SOD: Super Oxide Dimutese
VEGF: vascular endothlial growth factor
MDA: malonaldehyde
STAT3: signal transducer and activator of transcription 3
